# Glycemic control and diabetes complications among adult type 2 diabetic patients at public hospitals in Hadiya zone, Southern Ethiopia

**DOI:** 10.1371/journal.pone.0282962

**Published:** 2023-03-23

**Authors:** Abraham Lomboro Dimore, Zerihun Kura Edosa, Asmelash Abera Mitiku

**Affiliations:** 1 Department of Epidemiology, Faculty of Public Health, Institute of Health, Jimma University, Jimma, Ethiopia; 2 Disease Prevention and Control Directorate, Gambella Regional Health Bureau, Gambella, Ethiopia; CCMB: Centre for Cellular and Molecular Biology CSIR, INDIA

## Abstract

**Background:**

Diabetes is one of the biggest worldwide health emergencies of the 21^st^ century. A major goal in the management of diabetes is to prevent diabetic complications that occur as a result of poor glycemic control. Identification of factors contributing to poor glycemic control is key to institute suitable interventions for glycemic control and prevention of chronic complications.

**Methods:**

A hospital-based cross-sectional study was conducted among 305 adult type 2 diabetic patients at public hospitals in Hadiya zone from March 1–30, 2019. The study participants were selected by systematic sampling technique. Data were collected using a pretested structured questionnaire and patient chart review; anthropometric and blood pressure measurements were taken. Multivariable logistic regression analysis was used to identify factors associated with poor glycemic control. Adjusted odds ratios (AOR) with respective 95% Confidence Interval (CI) and p < 0.05 were used to set statistically significant variables.

**Results:**

Out of 305 diabetic patients, 222 (72.8%) were found to have poor glycemic control. Longer duration of diabetes (5–10 years) [AOR = 2.24, 95% CI: 1.17–4.27], lack of regular follow-up [AOR = 2.89, 95% CI: 1.08–7.71], low treatment adherence [AOR = 4.12, 95% CI: 1.20–8.70], use of other alternative treatments [AOR = 3.58, 95% CI: 1.24–10.36], unsatisfactory patient physician relationship [AOR = 2.27, 95% CI: 1.27–4.04], and insufficient physical activity [AOR = 4.14, 95% CI: 2.07–8.28] were found to be independent predictors of poor glycemic control. Diabetes Mellitus (DM) complications were slightly higher among participants with poor glycemic control (39.2%), duration of DM 10 and above years (41.9%), low medication adherence (48.5%), taking oral anti-diabetics (54.3%), and DM patients having unsatisfactory patient provider relationship (72.4%).

**Conclusion:**

A significant proportion of diabetic patients had poor glycemic control and DM complications. Therefore, appropriate interventions are required to maintain optimal glycemic control and prevent the development of life-threatening complications among DM patients.

## Introduction

Diabetes Mellitus (DM) refers to a group of common metabolic disorders that has a main characteristic feature of hyperglycemia [1]. Globally, an estimated 463 million people (9.3% of adults, 20–79 years) were living with diabetes in 2019. Its age-standardized prevalence increased by 62% within 10 years; from 285 million in 2009 to 463 million in 2019. The number of people living with diabetes was predicted to rise to 10.2% (578 million) by 2030 and 10.9% (700 million) by 2045 [2]. In 2019, an estimated more than four million adults died of diabetes and its complications (11.3% of all-cause mortality) [3].

The African region, where diabetes was once rare, has witnessed a surge in the condition. Its prevalence in this region among 20–79 years adults is 4.7%. According to global projections for 2030 and 2045, DM prevalence in the region is predicted to rise to 5.1% and 5.2%, respectively [2]. In 2019, there were around 366,227 deaths attributed to diabetes, with 73.1% of these deaths occurring in people under the age of 60 years, which was higher in proportion to any other region in the world [3].

In Ethiopia, diabetes prevalence is increasing among the adult population. According to the report from systematic review and meta-analysis the prevalence of DM in Ethiopia was 6.5% [4]. It is becoming a growing public health problem along with other non-communicable diseases in Ethiopia. Furthermore, its prevalence is also reported increasingly across different localities of the country, which is 0.3% for the lowest and 7.0% for the highest prevalence [5].

For successful control of risk resulting from long-term diabetic complications, optimal glycemic control is paramount. Deprived and insufficient glycemic control among patients with type 2 diabetes establishes a main public health problem and the foremost risk for the development of diabetic complications. Uncontrolled diabetes mellitus leads to micro-vascular and macro-vascular complications [1]. Furthermore, these complications due to poorly controlled diabetes are major causes of disability, premature death, and reduced quality of life [6].

Evidence in Ethiopia has reported that with increasing prevalence and related complications, diabetes is becoming a pressing public health problem [5]. Despite this alarming growth in the prevalence of diabetes, little has been studied regarding glycemic control status, related factors and DM complications. There is a gap and little information is available on these conditions in Ethiopia, particularly in this study area. Therefore, this study aimed to assess glycemic control and DM complications among adult type 2 diabetic patients at public hospitals in Hadiya zone, Southern Ethiopia.

## Materials and methods

### Study design, area, and study period

A facility-based cross-sectional study was conducted at public hospitals in Hadiya zone from March 1, 2019 to March 30, 2019. Hadiya zone is one of the administrative zones in Southern Nations, Nationalities, and Peoples Regional State (SNNPR). The Zone has four public hospitals (one teaching hospital and three primary hospitals). From these public hospitals, two (Nigist Ellen Mohammed Memorial and Shone primary Hospitals) of them provide chronic illness care for diabetic patients and there are 1,241 diabetic patients (56 type 1 and 1,185 type 2 DM). The hospitals do not have the glycated hemoglobin (HbA1c) test, but the fasting blood glucose of patients was measured based on their follow-up appointment.

### Population

All type 2 diabetic patients aged ≥ 18 years old on follow-up at Nigist Ellen Mohammed Memorial and Shone primary hospitals were a source population, and type 2 diabetic patients aged ≥ 18 years old who present during the study period and fulfilled the eligibility criteria were study population. Type 2 diabetic patients on ant-diabetic(s) treatment for at least six months and patients who had at least three consecutive blood glucose measurements in three months were included in this study. Patients with critical illness who were unable to communicate at the time of data collection, patients with hearing problems and previously diagnosed psychiatric illness and pregnant women with diabetes were excluded from the study.

### Sample size and sampling technique

The required sample was calculated using a single population proportion estimation formula considering the following assumptions: 59.2% prevalence of poor glycemic control from the study done in Shanan Gibe Hospital, Southwest Ethiopia [7], 95% confidence level (CI), 5% margin of error and 10% non-response rate. Since the source population was less than 10,000, considering the correction formula, the total calculated sample yielded 311.

A systematic random sampling technique was applied to recruit study participants. The diabetic clinic provides services three days per week and on average 92 type 2 diabetic patients are served per day at Nigist Ellen Mohammed Memorial Hospital. In Shone primary hospital, diabetic patients had two days per month for follow-up and on average 40 patients were served per day. The study participants were allocated for both hospitals by proportional to population size allocation. By dividing the total type 2 DM patients eligible (1,185) by the sample size required (311), which yields a sampling interval of four. Sample recruitment was performed concurrently in both hospitals. The first participant was selected by lottery method. Thus, every fourth patient coming to the clinic for a follow-up service was interviewed until the total sample size reached.

### Data collection procedure

Data were collected by using pretested structured questionnaires to capture information on socio-demographic and economic characteristics; clinical characteristics; knowledge about diabetes, and attitudes toward DM care; and adherence to diabetic self-care activities. A checklist was used to abstract data from the medical records. Sphygmomanometer, weight scale, and stadiometer were used to measure blood pressure, weight, and height, respectively.

### Measurements and operational definition

Fasting blood glucose readings of the last three diabetic clinic visits were obtained from patients’ medical records and computed mean fasting glucose levels. Poor glycemic control was operationally defined if the mean fasting glucose(FBG) level was above 130mg/dL [8].

Adherence to antidiabetic medications was measured by using Morisky Medication Adherence Scale (MMAS 8-item) [9]. The scale contains questions asking the patient to respond "Yes" or "No" to a set of eight questions. A positive response indicated a problem with medication adherence. Therefore, higher scores indicate that a patient has the least adherence to medications. For all questions, responses were coded 1 if patients responded "Yes" otherwise, 0 if not, except one question (Did you take all your medicines yesterday?) that was coded in reverse. The total score was computed and adherence was categorized as high, medium, and low if the participants score was 0, 1–2, and 3–8, respectively.

Patient-provider relationship was measured by using Patient Doctor Relationship Questionnaire (PDRQ_9) consisting of nine questions with a five-point Likert-type scale, where 1 = very inappropriate and 5 = very appropriate [10]. The total score was computed and participants who scored mean and above were considered to have a satisfactory patient-provider relationship.

Knowledge of patients about diabetes was assessed by using eight knowledge questions. Percentage out of total score was computed and participants who answered six (75%) questions out of total knowledge questions correctly were categorized as having good knowledge about diabetes. Attitude of patients towards diabetic care was assessed by using seven questions on a five-point Likert- type scale, where 1 = strongly disagree and 5 = strongly agree. Three items have been negatively worded, which requires reverse coding. Its internal consistency was checked by using reliability statistics with Cronbach’s α = 0.81 during the pretest. The total score was computed and patients were considered as having a positive attitude towards diabetic care if s/he scored mean and above for attitude questions.

Blood pressure was measured after the patient sat and rested for a few minutes with the arm held at a position that was around the heart. Blood pressure was measured twice and recorded from a mean of two measurements as per American Diabetes Association (ADA) recommendations [8]. Study participants whose systolic BP ≥ 140 mmHg and/ or diastolic BP ≥ 90 mmHg or current use of antihypertensive medication irrespective of the current BP were considered as hypertensive.

Anthropometric measurements were measured using standardized techniques and calibrated equipment. The weight of the participants was measured to the nearest 0.1 kg. The scale was placed on a hard surface and the participants were measured by wearing light clothing and bare feet. Height of the participants was measured to the nearest 0.5 cm using a stadiometer. Then, Body Mass Index (BMI) of the participants was calculated as weight in kg divided by height in meters squared and subjects were considered as normal (BMI = 18.5–24.9 kg/m^2^), overweight (BMI = 25–29 kg/m^2^) and obese (BMI ≥ 30 kg/m^2^) [8].

Diabetic self- care activities were assessed by using Summary of Diabetic Self-care Activity measure (SDSCA), which contains 11 items on diet, exercise, self- monitoring of blood glucose, foot care, and cigarette smoking [11]. Exercise was measured based on response to items five and six, then participants who participated in at least 30 minutes of physical activity for 3 or more days or participated in specific exercise session during the last seven days were categorized as having adequate adherence to exercise.

The study participants who used other non-medical treatment options like traditional or herbal medicines and religious healing practices for the treatment of diabetes were considered as having used other alternative treatments. A diabetic patient who visited the diabetic clinic based on appointment regularly within the previous six months was considered to having regular follow-up at the diabetic clinic.

### Data management and quality assurance

The questionnaire and checklist were translated from English language to Amharic and Hadiyissa (local language) and translated back to English language to check its consistency. One-day training was given for data collectors and supervisors on the objectives, process of data collection, and how to take anthropometric measurements. Pretest was done on 5% of the sample size in order to check the clarity and internal consistency of the questionnaire and checklist prior to the actual data collection.

The equipment for measuring weight, height, and blood pressure were calibrated to the standard before measuring each participant. Completeness, accuracy, clarity, and consistency of data were checked daily after data collection time by supervisors. The overall activities were monitored by the principal investigators. Finally, the collected data were entered into a computer using epidata3.1 version software.

### Statistical analysis

The analysis was done in Statistical Package for the Social Science (SPSS) 20 version software. Descriptive statistics including mean (standard deviation), median (inter-quartile range) and range values for continuous variables; and percentage and frequency tables for categorical variables were employed. Normality assumption was checked for continuous variables.

Bivariate analysis was employed to determine the presence of an association between poor glycemic control and each independent variable using binary logistic regression. Variables that were found significant at p-value less than 0.25 in bivariate analysis were selected as candidate variables for multivariable analysis.

Multivariable analysis was carried out to identify independent predictors of poor glycemic control and to control for confounders. Backward stepwise logistic regression was used to determine independent predictors with P-value less than 0.05 with their respective AOR and 95% CI. The model fitness was tested by using Hosmer and Lemeshow goodness of fit test and was declared fit.

### Ethical considerations

The study was approved by Institutional Review Board (IRB) of Institute of Health Sciences at Jimma University; Southwest Ethiopia. Permission to conduct the study was obtained from both hospital administrative offices. We have informed the participants about the objectives of the study, the procedures, and their voluntary participation in the study before conducting the interviews. Then, we obtained informed verbal consent from each participant and it was documented on each participant questionnaire. Data were collected anonymously to ensure confidentiality. Moreover, individual counseling on self-care practices was given for participants with poor glycemic control to maximize the benefits of the study.

## Results

### Socio-demographic characteristics of the respondents

A total of three hundred and five type two diabetes patients participated in this study with a response rate of 98%. Out of the total participants, 182 (59.7%) were males and the median (IQR) age of the respondents was 44 (19) years, ranging from 19 to 78 years. Nearly half (47.5%) of them were within the age category of 40–60 years. Three fifths (60%) of the respondents were following Protestant religion and four out of five (83.9%) respondents were married. Nearly one third 96 (31.5%) of the respondents attained college and above; 105 (34.4%) were government employees and 212 (69.5%) were urban residents **(Table 1)**.

**Table 1:** Socio-demographic and economic characteristics of the study participants attending diabetic clinic at public hospitals in Hadiya zone, Southern Ethiopia, 2019.

Characteristics(N = 305)	Categories	Number (%)
Sex	Male	182(59.7)
	Female	123(40.3)
Age Median(IRQ) = 44(19)	< 40	111(36.4)
	40–60	145(47.5)
	> 60	49(16.1)
Religion	Protestant	183(60)
	Orthodox	79(25.9)
	Muslim	25(8.2)
	Catholic	18(5.9)
Marital status	Single	21(6.9)
	Married	256(83.9)
	Divorced/widowed	28(9.2)
Educational status	Unable to read and write	72(23.6)
	Able to read and write	81(26.6)
	Primary school (1–8 grade)	27(8.9)
	Secondary school(9–12 grade)	29(9.5)
	College & above	96(31.5)
Occupational status	Government employee	105(34.4)
	Merchant	71(23.3)
	Housewife	59(19.3)
	Farmer	52(17.0)
	Others^**a**^	18(5.9)
Residence	Urban	212(69.5)
	Rural	93(30.5)
Family income	< 3500(ETB)	97(31.8)
	≥ 3500(ETB)	208(68.2)

^**a**^student, retired

### Glycemic control status

Fasting blood glucose readings of the last three diabetic clinic visits were obtained from patients’ medical records. Mean fasting blood glucose (FBG) measurements of the last three months were used to determine glycemic control. The mean (SD) FBG level of the participants was 167.49 (58.183) mg/dL. The minimum and maximum FBG measurements were 90 mg/dL and 478 mg/dL, respectively. The prevalence of poor glycemic control was 72.8% (95% CI: 67.8% -78.1%). The prevalence of poor glycemic control was 72.8% (95% CI: 67.8% -78.1%). Poor glycemic control was higher among government employees (77 [73.3%]), age category 40–60 years (106 [73.1%]), married (186 [72.7%]), male (127 [69.8%]), urban dwellers (146 [68.9%]), and those who attained college and above educational level (65 [67.7%]) (**S1 Table**).

### Diabetes complications

Diabetes-related complications were found in 105 (34.4%) of the study participants. The common DM complication among the study participants were retinopathy (26.7%), foot ulcer (17.1%), nephropathy (14.3%), and neuropathy (10.5%) (**S2 Table**). The prevalence of DM complications was predominant among participants with duration of 10 and above years (44 [41.9%]), low medication adherence (51 [48.5%]), taking oral anti-diabetics (57 [54.3%]), and DM patients having unsatisfactory patient provider relationship (76 [72.4%]) **(Table 2)**. Likewise, prevalence of DM complications was higher among patients with poor glycemic control 87 (39.2%) than those with good glycemic control.

**Table 2:** Clinical and anthropometric characteristics of the study participants attending diabetic clinic at public hospitals in Hadiya zone, Southern Ethiopia, 2019.

Variables	Category	Glycemic control		DM complications	
		Poor N (%)	Good N (%)	Yes N (%)	No N (%)
Family history of DM	No	143 (27.0)	53 (73.0)	68 (64.8)	128 (64.0)
	Yes	79 (72.5)	30 (27.5)	37 (35.2)	72 (36.0)
Family support	No	38 (71.7)	15 (28.3)	10 (10.5)	43 (21.5)
	Yes	184 (73.0)	68 (27)	95 (89.5)	157 (78.5)
Duration of diabetes	<5 years	86 (64.7)	47 (35.3)	31 (29.5)	102 (51.0)
	5–10 years	86 (79.6)	22 (20.4)	30 (28.6)	78 (39.0)
	≥ 10 years	50 (78.1)	14 (21.9)	44 (41.9)	20 (10.0)
Co morbidity	No	153 (70.2)	65 (29.8)	52 (49.5)	166 (83.0)
	Yes	69 (79.3)	18 (20.7)	53 (50.5)	34 (17.0)
Type of anti-diabetics	Insulin only	77 (74.8)	26 (25.2)	34 (32.4)	69 (34.5)
	Oral medication	130 (72.6)	49 (27.4)	57 (54.3)	122 (61.0)
	Insulin and oral	15 (65.2)	8 (34.8)	14 (13.3)	9 (4.5)
Regular follow up	No	40 (87.0)	6 (13.0)	16 (15.2)	30 (15.0)
	Yes	182 (70.3)	77 (29.7)	89 (84.8)	170 (85.0)
Counseling	No	72 (75.8)	23 (24.2)	34 (32.4)	61 (30.5)
	Yes	150 (71.4)	60 (28.6)	71 (67.6)	139 (69.5)
use of other alternative treatments	No	177 (69.4)	78 (30.6)	77 (73.3)	178 (89.0)
	Yes	45 (90.0)	5 (10.0)	28 (26.7)	22 (11.0)
Patient provider relationship	Satisfactory	76 (64.4)	42 (35.6)	29 (27.6)	89 (44.5)
	Unsatisfactory	146 (78.1)	41 (21.9)	76 (72.4)	111 (55.5)
Body mass index	Normal	146 (75.3)	48 (24.7)	67 (63.8)	127 (63.5)
	Overweight	76 (68.5)	35 (31.5)	38 (36.2)	73 (36.5)
Blood pressure	Normal	145 (69.4)	64 (30.6)	52 (49.5)	157 (78.5)
	Hypertensive	77 (80.2)	19 (19.8)	53 (50.5)	43 (21.5)
Medication adherence	High adherence	88 (62.4)	53 (37.6)	32 (30.5)	109 (54.5)
	Moderate adherence	41 (69.5)	18 (30.5)	22 (21.0)	37 (18.5)
	Low adherence	93 (88.6)	12 (11.4)	51 (48.5)	54 (27)

### Clinical characteristics of T2 DM patients

The median (IQR) diabetes duration of the participants was 5 (5) years and 133 (43.6%) of the participants had duration of less than five years. Out of total participants, 87 (28.5%) of them had other chronic diseases and 105 (34.4%) of the respondents had diabetes-related complications that were previously diagnosed. Diabetic retinopathy was the most common DM complication, accounting for 73.3% (**S2 Table**).

Of the total respondents, 50 (16.4%) of them use other alternative treatments for diabetes, of which 44 (88%) use traditional medicine and six (12%) use religious healing practices (**S2 Table**). Three-fifths (61.3%) of the participants had an unsatisfactory patient-provider relationship and 46 (15.1%) of them did not have regular follow-up at the diabetic clinic within the previous six months. Regarding medication adherence, 105 (34.4%) of the respondents had low adherence **(Table 2)**.

The mean (SD) BMI of the respondents was 24.18 (2.76) Kg/m^2^ and 36.4% of the respondents had overweight. The mean (SD) systolic and diastolic BP was 131.92 (16.42) and 84.72 (7.76) mmHg, respectively. Out of total participants, about 96 (31.5%) of them were hypertensive **(Table 2)**.

### Knowledge and attitude towards diabetic care

Of the total participants, 139 (45.6%) had poor knowledge about diabetes and the rest had good knowledge. The mean score for attitude is 28.21 (±3.079) with a minimum score of 17 and a maximum score of 35. Nearly half (48.9%) of the respondents had a negative attitude towards diabetic care.

### Factors associated with glycemic control

Bivariate analysis was done to see the association between the independent variables and poor glycemic control. According to bivariate analysis; sex, educational status, marital status, residence, income, duration of diabetes, comorbidity, regular follow-up, use of other alternative treatments, patient-provider relationship, medication adherence, knowledge, attitude, blood pressure, body mass index, and physical activity showed association with poor glycemic control at P-value less than 0.25 (**S3 and S4 Tables**). These variables were entered into multivariable analysis to determine independent predictors of poor glycemic control.

In multiple logistic regression, a statistically significant difference was found in poor glycemic control due to duration of diabetes, follow-up to DM clinic, medication adherence, using other alternative treatments, unsatisfactory patient-provider relations and insufficient physical activity **(Table 3)**.

**Table 3:** Multivariable analysis of factors associated with poor glycemic control among T2 DM adult patients at Public hospital in Hadiya Zone, Southern Ethiopia, 2019.

Variables (n = 305)	Category	Glycemic control, n (%)		COR (95% CI)	AOR (95% CI)	P -value
		Poor (n = 222)	Good (n = 83)			
Sex	male	127	55	1	1	
	female	95	28	1.47(0.87–2.49)	1.25(0.66–2.38)	0.491
Marital status	single	13	8	1	1	
	Married	186	70	1.64(0.65–4.14)	1.46(0.47–0.52)	0.512
	Divorced/widowed	23	5	2.83(0.77–10.47)	0.96(0.18–4.96)	0.967
Educational status	Unable to read and write	63	9	3.34(1.47–7.57)	0.98(0.33–2.92)	0.973
	Able to read and write	60	21	1.36(0.71–2.63)	0.84(0.38–1.81)	0.649
	Primary school	16	11	0.69(0.29–1.67)	0.47(0.16–1.32)	0.153
	Secondary school	18	11	0.78(0.33–1.85)	0.54(0.21–1.40)	0.206
	College and above	65	31	1	1	
Residence	Urban	146	66	1	1	
	Rural	76	17	2.02(1.11–3.69)	1.40(0.67–2.93)	0.367
Family income	< 3500(ETB)	78	19	1.83(1.02–3.26)	1.02(0.42–2.47)	0.959
	≥ 3500(ETB)	144	64	1	1	
Comorbidity	No	153	65	1	1	
	Yes	69	18	1.63(0.90–2.95)	0.40(0.13–1.25)	0.115
Duration of diabetes	< 5 years	86(38.7)	47(56.6)	1	1	
	5–10 years	86(38.7)	22(26.5)	2.14(1.19–3.85)	2.24(1.17–4.27)	0.014*
	> = 10 years	50(22.5)	14(16.9)	1.95(0.98–3.90)	1.40(0.64–3.06)	0.395
Regular follow up	No	40(18.1)	6(7.2)	2.82(1.15–6.93)	2.89(1.08–7.71)	0.035*
	Yes	182(81.9)	77(92.8)	1	1	
Blood pressure	Normal	145	64	1	1	
	Hypertensive	77	19	1.79(1.00–3.20)	1.31(0.62–2.77)	0.478
BMI	Normal	146	48	1	1	
	Overweight	76	35	0.71(0.43–1.20)	0.87(0.48–1.58)	0.641
Medication adherence	High	88(39.6)	53(63.9)	1	1	
	Moderate	41(18.5)	18(21.7)	1.37(0.72–2.63)	1.57(0.77–3.21)	0.220
	Low	93(41.9)	12(14.5)	4.67(2.34–9.32)	4.12(1.20–8.70)	<0.001*
Use of other alternative treatments	No	177(79.7)	78(94.0)	1	1	
	Yes	45(20.3)	5(6.0)	3.97(1.52–10.37)	3.58(1.24–10.36)	0.018*
Patient provider relation	satisfactory	76(34.2)	42(50.6)	1	1	
	Unsatisfactory	146(65.8)	41(49.4)	1.97(1.18–3.28)	2.27(1.27–4.04)	0.005*
Knowledge about DM	Good	110	56	1	1	
	poor	112	27	2.11(1.24–3.59)	0.98(0.51–1.90)	0.970
Attitude towards DM care	Positive	99	57	1		
	Negative	123	26	2.72(1.60–4.65)	1.65(0.89–3.08)	0.114
Physical exercise	Adequate	28(12.6)	26(31.3)	1	1	
	Inadequate	194(87.4)	57(68.7)	3.16(1.72–5.82)	4.14(2.07–8.28)	<0.001*

AOR: Adjusted Odds Ratio; COR: Crude Odds Ratio

* statistically significant at P-value < 0.05

## Discussion

It is an established fact that diabetes can cause complications in those patients whose blood glucose level is not controlled [1]. The main goal of diabetes management is to ensure optimal glycemic control to delay and prevent complications. This study assessed the prevalence of poor glycemic control and its associated factors among type two diabetic patients.

The findings of this study showed that nearly three-fourths (72.8%) of diabetic patients in the study area had poor glycemic control. This finding was comparable with earlier studies done in Saudi Arabia (74.9%) [12], Tanzania (69.7%) [13], Dessei, Northeast Ethiopia (70.8%) [14], and Jimma, Southwest Ethiopia (70.9%) [15]. However, it is ahigher prevalence than that of studies which reported 64.9% in Nekemte referral Hospital and 59.2% in Shanan Gibe Hospital, Southwest Ethiopia [7, 16]. The possible reason for this high prevalence of poor glycemic control could be the clinical characteristics of the patients, low medication adherence, and insufficient physical activity of the patients in the current study. This finding was lower than a study done in Tikur Anbesa specialized Hospital (TASH), Ethiopia, which reported 80% [17] of the study participants had poor glycemic control. The possible explanation for this difference could be that patients seeking advanced management were referred to TASH and patients from the whole region of the country were referred to TASH [17]. The results of the current study highlight the need to work more on the optimal management of diabetes, since maintaining the recommended glycemic level is the main therapeutic goal for all patients with diabetes.

The current study showed that longer duration of diabetes is significantly associated with poor glycemic control. This finding is consistent with other similar studies [12, 14, 17–19]. However, this finding is slightly lower in strength of association than the finding from a study done in Shanan Gibe Hospital [7]. The possible reason for this difference could be the majority (43.6%) of the patients in the current study had short duration (less than five years) of diabetes, while in that one 49.4% of the participants had long duration (greater than 10 years) of diabetes. The possible explanation for this finding could be due to progressive impairment of insulin secretion over time because of the failure of β-cells and increased insulin resistance to control blood sugar [20]. Moreover, it might be due to difficulty for the patients to continue monitoring of blood glucose level and adjust with the treatment, exercise and diet [21, 22]. Therefore, measures should be put in place for education for diabetes patients, emphasizing more on self-care activities, especially for patients with long duration of diabetes.

In the current study, a lack of regular follow-up was significantly associated with poor glycemic control. This finding is in agreement with previous studies done in Brazil and Southwest Ethiopia [7, 23]. The possible reason for this finding could be that patients who are not regularly following the diabetic clinic might be noncompliant to diabetic self-care activities and treatment [24, 25]. In addition, those patients who are not regularly following the diabetic clinic might not know their blood sugar level and they might not get counseling about their disease condition. This finding implies that health care providers should give attention to encourage the patients to visit the diabetic clinic regularly.

In this study, poor glycemic control appeared to be greater among patients who had low medication adherence compared with high adherence. This finding is comparable with other studies conducted in Jimma and Gondar hospitals [15, 26]. However, the current finding is higher in strength of association than the finding from a study done in Tripoli, Libya [27]. The reason for this difference might be due to the different measurement scores in these two studies. The possible explanation for this finding is that low adherence to treatment is one of the barriers that prevents many diabetic patients from achieving optimal glycemic levels [28]. Furthermore, it might be due to lack of patients’ knowledge about the importance of treatment adherence, which results in better glycemic control. The finding implies that health facilities should consider developing educational programs that emphasize life-style modification with the importance of adherence to treatment would be of great benefit for optimal glycemic control. Moreover, health care providers should discuss barriers to treatment adherence when counseling patients and solutions should be tailored toward individual needs.

In the present study, use of other alternative treatments (traditional medicine and religious healing practices) is significantly associated with poor glycemic control. Patients who used other alternative treatments were more likely to have poor glycemic control. This finding is supported by a systematic review of literature in Sub-Sahara African countries in which the use of herbal medicines and traditional healers was frequently mentioned, although it is not part of the ADA self-management guidelines [29]. A study in Northern Ethiopia also revealed that the majority (62%) of diabetes patients were herbal medicine users and most (87.1%) of them did not consult their physicians about their herbal medicine use [30]. This finding could be due to the fact that patients who used other alternative treatments might be low medication adherent and this might be leading to poor glycemic control [28]. Thus, health care providers should consult patients regarding use of other alternative treatments and encourage them to adhere to prescribed medication.

Having an unsatisfactory patient-provider relationship was found to be an independent predictor of poor glycemic control among type 2 diabetic patients. The possible reason could be those patients who have a satisfactory patient-physician relationship might be well encouraged to act in accordance with self-care activities. The finding implies that health care providers should pay attention to developing effective patient-provider relation and communication skills when counseling diabetic patients.

The current study also revealed that patients with insufficient physical activity had poor glycemic control, which is consistent with prior studies done in Tripoli, Libya, and Jimma, Southwest Ethiopia [27, 31]. Nevertheless, it is lower than the finding from the study done in Saudi Arabia [12]. The variation could be due to that the previous study measured physical activity at least 30 minutes for three days per week, while the current study measured physical activity by mean score for physical exercise done in the last seven days using the SDSCA tool. The possible explanation for this finding might be due to having inadequate knowledge about the benefits of regular physical exercise and a fear of hypoglycemia. This implies that encouraging diabetic patients to do physical exercise is a crucial part of diabetes education for optimal glycemic control. Furthermore, physical exercise has not only been reported to raise glycemic control, but also to improve a patients insulin sensitivity and to repair some of the damage caused by diabetes-associated complications, such as impaired cardiovascular health, one of the most common complications [32].

Prevalence of diabetic complications in this study was slightly higher among diabetic patients having poor glycemic control (87 [39.2%]) compared to their counterparts. A study done in Gondar Ethiopia, also found diabetic complications were higher among DM patients with poor glycemic control [33]. The commonest diabetic complication identified in this study was retinopathy (26.7%). Likewise, a case-control study conducted in Brazil revealed that retinopathy is predominant among DM patients with poor glycemic control [34]. Moreover, a follow-up study in the USA showed the association between the level of glucose and diabetic retinopathy, which indicated that controlling blood glucose level using rigorous treatment gave rise to delayed slow progression of diabetic retinopathy [35].

The lack of a relationship between educational status and poor glycemic control in this study is not consistent with the findings of previous studies [14, 15, 18, 36], which reported that no formal education was associated with poor glycemic control. The reason for this difference could be that the majority of patients in previous studies had no formal education, while in the current study, the majority (31.5%) of the patients had attained college and above. In addition to this, type of treatment (being on insulin treatment) does not show significant association with poor glycemic, which is not in line with studies done previously elsewhere [17, 26, 27, 37, 38]. This might be due to the majority (58.7%) of the patients in the current study were taking oral anti-diabetics. The other reason could be that type 2 diabetes patients are treated by insulin when their blood glucose level is not controlled by oral anti-diabetics.

### Limitations of the study

The current study has its own limitations that should be acknowledged. The use of mean FBG over HbA1c is one limitation; thus possibly under estimate the prevalence of poor glycemic control. However, an effort was made to overcome this issue by taking the mean average of the last three consecutive visits for FBG measurements. In addition, the incompleteness of the patients’ charts is one of the shortcomings of this study since some items like co-morbidities were abstracted from the patient charts. Furthermore, the subjective nature of self-reported responses for some items might be limited by recall bias, and since the data collectors were health professionals, social desirability bias may also occur for some items.

## Conclusion

The current study revealed that the prevalence of poor glycemic control and diabetic complications is noticeably high among diabetes patients. DM complications were found slightly higher among patients with poor glycemic control. Poor glycemic control showed significant association with longer duration of diabetes, lack of regular follow-up, low adherence to treatment, use of other alternative treatments, unsatisfactory patient-physician relations, and insufficient physical exercise. Therefore, we recommend considering developing educational programs that emphasize the importance of medication adherence. Regular follow-up and physical activity would be of great benefit in poor glycemic control. It is also paramount to enhance effective patient-provider relations and communication skills when counseling diabetic patients. Furthermore, considering consulting the patients regarding the use of other alternative treatments at each visit is also essential.

## Supporting information

S1 Table Glycemic control status and socio-demographic characteristics of the study participants attending diabetic clinic at public hospitals in Hadiya zone, Southern Ethiopia, 2019. https://doi.org/10.6084/m9.figshare.20449119.
(DOCX)

S2 Table Diabetic complications and use of other alternative treatment among study participants attending diabetic clinic at public hospitals in Hadiya zone, Southern Ethiopia, 2019. https://doi.org/10.6084/m9.figshare.20449296.
(DOCX)

S3 Table Bivariate analysis of socio-demographic and economic factors among type 2 diabetic patients at Public hospitals in Hadiya Zone, Southern Ethiopia, 2019. https://doi.org/10.6084/m9.figshare.20449344.
(DOCX)

S4 Table Bivariate analysis of clinical factors among T2 DM adult out patients at public Hospitals in Hadiya Zone, Southern Ethiopia, 2019. https://doi.org/10.6084/m9.figshare.20449371.
(DOCX)
