# Supplementary material for: Glycemic control and diabetes complications among adult type 2 diabetic patients at public hospitals in Hadiya zone, Southern Ethiopia
Source: PLoS One. 2023 Mar 23;18(3):e0282962. doi: 10.1371/journal.pone.0282962 (PMC10035868; doi:10.1371/journal.pone.0282962)
Supplement: S1 Table — https://doi.org/10.6084/m9.figshare.20449119. (DOCX) [file pone.0282962.s001.docx]

S1 Table. Glycemic control status and socio-demographic characteristics of the study participants attending diabetic clinic at public hospitals in Hadiya zone, Southern Ethiopia, 2019.

| **Variable** | **Category** | **Glycemic control** | |
| --- | --- | --- | --- |
|  |  | **Poor (n=222), N (%)** | **Good (83), N (%)** |
| Sex | Male | 127 (69.8) | 55 (30.2) |
|  | Female | 95 (77.2) | 28 (22.8) |
| Age, years | <40 | 78(70.3) | 33(29.7) |
|  | 40-60 | 106(73.1) | 39(26.9) |
|  | >=60 | 38(77.6) | 11(22.4) |
| Marital status | Single | 13(61.9) | 8(38.1) |
|  | Married | 186(72.7) | 70(27.3) |
|  | Divorced/widowed | 23(82.1) | 5(17.9) |
| Educational status | Unable to read and write | 63(87.5) | 9(12.5) |
|  | Able to read and write | 60(74.1) | 21(25.9) |
|  | Primary school | 16(59.3) | 11(40.7) |
|  | Secondary school | 18(62.1) | 11(37.9) |
|  | College and above | 65(67.7) | 31(32.3) |
| Occupational status | Government employee | 77(73.3) | 28(26.7) |
|  | Merchant | 48(67.6) | 23(32.4) |
|  | Housewife | 47(79.7) | 12(20.3) |
|  | Farmer | 38(73.1) | 14(26.9) |
|  | Others | 12(66.7) | 6(33.3) |
| Residence | Urban | 146(68.9) | 66(31.1) |
|  | Rural | 76(81.7) | 17(18.3) |
| Family income | <3500(ETB) | 78(80.4) | 19(19.6) |
|  | >=3500(ETB) | 144(69.2) | 64(30.8) |
