# Supplementary material for: Glycemic control and diabetes complications among adult type 2 diabetic patients at public hospitals in Hadiya zone, Southern Ethiopia
Source: PLoS One. 2023 Mar 23;18(3):e0282962. doi: 10.1371/journal.pone.0282962 (PMC10035868; doi:10.1371/journal.pone.0282962)
Supplement: S2 Table — https://doi.org/10.6084/m9.figshare.20449296. (DOCX) [file pone.0282962.s002.docx]

S2 Table. Diabetic complications and use of other alternative treatment among study participants attending diabetic clinic at public hospitals in Hadiya zone, Southern Ethiopia, 2019.

| **Characteristics** | **Category** | **Frequency** | **Percentage** |
| --- | --- | --- | --- |
| Diabetic complication | No | 200 | 65.6 |
|  | Yes | 105 | 34.4 |
| Retinopathy | No | 28 | 26.7 |
|  | Yes | 77 | 73.3 |
| Neuropathy | No | 94 | 89.5 |
|  | Yes | 11 | 10.5 |
| Nephropathy | No | 90 | 85.7 |
|  | Yes | 15 | 14.3 |
| Foot ulcer or amputation | No | 87 | 82.9 |
|  | Yes | 18 | 17.1 |
| Use of other alternative treatment | No | 255 | 83.6 |
|  | Yes | 50 | 16.4 |
| Use of traditional medicines | No | 6 | 12 |
|  | Yes | 44 | 88 |
| Use of religious healing practices | No | 44 | 88 |
|  | Yes | 6 | 12 |
