# Supplementary material for: Glycemic control and diabetes complications among adult type 2 diabetic patients at public hospitals in Hadiya zone, Southern Ethiopia
Source: PLoS One. 2023 Mar 23;18(3):e0282962. doi: 10.1371/journal.pone.0282962 (PMC10035868; doi:10.1371/journal.pone.0282962)
Supplement: S3 Table — https://doi.org/10.6084/m9.figshare.20449344. (DOCX) [file pone.0282962.s003.docx]

S3 Table. Bivariate analysis of socio-demographic and economic factors among type 2 diabetic patients at Public hospitals in Hadiya Zone, Southern Ethiopia, 2019.

| **Characteristics** | **Categories** | **Number**  **(%)** | **Glycemic control** | | **Crude OR (95% CI)** | **P-value** |
| --- | --- | --- | --- | --- | --- | --- |
|  |  |  | **Poor (n=222)** | **Good (83)** |  |  |
| Sex | male | 182(59.7) | 127 | 55 | 1 |  |
|  | female | 123(40.3) | 95 | 28 | 1.47[0.87-2.49] | 0.152* |
| Age | < 40 | 111(36.4) | 78 | 33 | 1 |  |
|  | 40-60 | 145(47.5) | 106 | 39 | 1.15[0.67-1.20] | 0.617 |
|  | > 60 | 49(16.1) | 38 | 11 | 1.46[0.67-3.20] | 0.343 |
| Marital status | single | 21(6.9) | 13 | 8 | 1 |  |
|  | Married | 256(83.9) | 186 | 70 | 1.64[0.65-4.14] | 0.296 |
|  | Divorced/widowed | 28(9.2) | 23 | 5 | 2.83[0.77-10.47] | 0.119* |
| Educational status | Unable to read and write | 72(23.6) | 63 | 9 | 3.34[1.47-7.57] | 0.004* |
|  | Able to read and write | 81(26.6) | 60 | 21 | 1.36[0.71-2.63] | 0.355 |
|  | Primary school  (1-8 grade) | 27(8.9) | 16 | 11 | 0.69[0.29-1.67] | 0.415 |
|  | Secondary school  (9-12 grade) | 29(9.5) | 18 | 11 | 0.78[0.33-1.85] | 0.574 |
|  | College & above | 96(31.5) | 65 | 31 | 1 |  |
| Occupational status | Government employee | 105(34.4) | 77 | 28 | 1 |  |
|  | Merchant | 71(23.3) | 48 | 23 | 0.76[0.39-1.47] | 0.412 |
|  | Housewife | 59(19.3) | 47 | 12 | 1.42[0.66-3.07] | 0.366 |
|  | Farmer | 52(17.0) | 38 | 14 | 0.98[0.47-2.09] | 0.973 |
|  | Others**^a^** | 18(5.9) | 12 | 6 | 0.73[0.25-2.12] | 0.560 |
| Residence | Urban | 212(69.5) | 146 | 66 | 1 |  |
|  | Rural | 93(30.5) | 76 | 17 | 2.02[1.11-3.69] | 0.022* |
| Family income | < 3500(ETB) | 97(31.8) | 78 | 19 | 1.83[1.02-3.26] | 0.043* |
|  | ≥ 3500(ETB) | 208(68.2) | 144 | 64 | 1 |  |

**^a^**student, retired; OR, Odds Ratio; * Statistically significant at P -value < 0.25
