# Supplementary material for: Glycemic control and diabetes complications among adult type 2 diabetic patients at public hospitals in Hadiya zone, Southern Ethiopia
Source: PLoS One. 2023 Mar 23;18(3):e0282962. doi: 10.1371/journal.pone.0282962 (PMC10035868; doi:10.1371/journal.pone.0282962)
Supplement: S4 Table — https://doi.org/10.6084/m9.figshare.20449371. (DOCX) [file pone.0282962.s004.docx]

S4 Table. Bivariate analysis of clinical factors among T2 diabetic patients at public Hospitals in Hadiya Zone, Southern Ethiopia, 2019.

| **Variables** | **Categories** | **Number**  **(%)** | **Glycemic control** | | **COR (95% CI)** | **P-value** |
| --- | --- | --- | --- | --- | --- | --- |
|  |  |  | **Poor (n=222)** | **Good (n=83)** |  |  |
| Family history of DM | No | 196(64.3) | 143 | 53 | 1 |  |
|  | Yes | 109(35.7) | 79 | 30 | 0.98[0.58-1.65] | 0.928 |
| Family support | No | 53(17.4) | 38 | 15 | 0.94[0.48-1.81] | 0.845 |
|  | Yes | 152(82.6) | 184 | 68 | 1 |  |
| Duration of diabetes | <5 years | 133(43.6) | 86 | 47 | 1 |  |
|  | 5-10 years | 108(35.4) | 86 | 22 | 2.14[1.19-3.85] | 0.011* |
|  | ≥ 10 years | 64(21.0) | 50 | 14 | 1.95[0.98-3.90] | 0.058* |
| Co morbidity | No | 218(71.5) | 153 | 65 | 1 |  |
|  | Yes | 87(28.5) | 69 | 18 | 1.63[0.90-2.95] | 0.108* |
| Complications | No | 200(65.6) | 135 | 65 | 1 |  |
|  | Yes | 105(34.4) | 87 | 18 | 2.33[1.29-4.19] | 0.004* |
| Type of anti diabetics | Insulin only | 103(33.8) | 77 | 26 | 1 |  |
|  | Oral medication | 179(58.7) | 130 | 49 | 0.90[0.52-1.56] | 0.697 |
|  | Insulin and oral | 23(7.5) | 15 | 8 | 0.63[0.24-1.66] | 0.354 |
| Regular follow up | No | 46(15.1) | 40 | 6 | 2.82[1.15-6.93] | 0.024* |
|  | Yes | 259(84.9) | 182 | 77 | 1 |  |
| Counseling | No | 95(31.1) | 72 | 23 | 1.25[0.72-2.19] | 0.429 |
|  | Yes | 210(68.9) | 150 | 60 | 1 |  |
| use of other alternative treatments | No | 255(83.6) | 177 | 78 | 1 |  |
|  | Yes | 50(16.4) | 45 | 5 | 3.97[1.52-10.37] | 0.005* |
| Patient provider relation | Satisfactory | 118(38.7) | 76 | 42 | 1 |  |
|  | Unsatisfactory | 187(61.3) | 146 | 41 | 1.97[1.18-3.28] | 0.009* |
| Body mass index | Normal | 194(63.6) | 146 | 48 | 1 |  |
|  | Overweight | 111(36.4) | 76 | 35 | 0.71[0.43-1.20] | 0.200* |
| Blood pressure | Normal | 210(68.5) | 145 | 64 | 1 |  |
|  | Hypertensive | 95(31.5) | 77 | 19 | 1.79[1.00-3.20] | 0.048* |
| Medication adherence | High adherence | 141(46.2) | 88 | 53 | 1 |  |
|  | Moderate adherence | 59(19.3) | 41 | 18 | 1.37[0.72-2.63] | 0.341 |
|  | Low adherence | 105(34.4) | 93 | 12 | 4.67[2.34-9.32] | <0.001* |

COR, Crude Odds Ratio; * statistically significant at P -value < 0.25
